# Appraising psychotherapy case studies in practice-based evidence: introducing Case Study Evaluation-tool (CaSE)

**DOI:** 10.1186/s41155-021-00175-y

**Published:** 2021-03-19

**Authors:** Greta Kaluzeviciute

**Affiliations:** Department of Psychosocial and Psychoanalytic Studies, University of Essex, Wivenhoe Park, Colchester, CO4 3SQ UK

**Keywords:** Systematic case studies, Psychotherapy research, Research appraisal tool, Evidence-based practice, Practice-based evidence, Research validity

## Abstract

Systematic case studies are often placed at the low end of evidence-based practice (EBP) due to lack of critical appraisal. This paper seeks to attend to this research gap by introducing a novel Case Study Evaluation-tool (CaSE). First, issues around knowledge generation and validity are assessed in both EBP and practice-based evidence (PBE) paradigms. Although systematic case studies are more aligned with PBE paradigm, the paper argues for a complimentary, third way approach between the two paradigms and their ‘exemplary’ methodologies: case studies and randomised controlled trials (RCTs). Second, the paper argues that all forms of research can produce ‘valid evidence’ but the validity itself needs to be assessed against each specific research method and purpose. Existing appraisal tools for qualitative research (JBI, CASP, ETQS) are shown to have limited relevance for the appraisal of systematic case studies through a comparative tool assessment. Third, the paper develops purpose-oriented evaluation criteria for systematic case studies through CaSE Checklist for Essential Components in Systematic Case Studies and CaSE Purpose-based Evaluative Framework for Systematic Case Studies. The checklist approach aids reviewers in assessing the presence or absence of essential case study components (internal validity). The framework approach aims to assess the effectiveness of each case against its set out research objectives and aims (external validity), based on different systematic case study purposes in psychotherapy. Finally, the paper demonstrates the application of the tool with a case example and notes further research trajectories for the development of CaSE tool.

## Introduction

Due to growing demands of evidence-based practice, standardised research assessment and appraisal tools have become common in healthcare and clinical treatment (Hannes, Lockwood, & Pearson, 2010; Hartling, Chisholm, Thomson, & Dryden, 2012; Katrak, Bialocerkowski, Massy-Westropp, Kumar, & Grimmer, 2004). This allows researchers to *critically appraise* research findings on the basis of their validity, results, and usefulness (Hill & Spittlehouse, 2003). Despite the upsurge of critical appraisal in qualitative research (Williams, Boylan, & Nunan, 2019), there are no assessment or appraisal tools designed for psychotherapy case studies.

Although not without controversies (Michels, 2000), case studies remain central to the investigation of psychotherapy processes (Midgley, 2006; Willemsen, Della Rosa, & Kegerreis, 2017). This is particularly true of systematic case studies, the most common form of case study in contemporary psychotherapy research (Davison & Lazarus, 2007; McLeod & Elliott, 2011).

Unlike the classic clinical case study, systematic cases usually involve a team of researchers, who gather data from multiple different sources (e.g., questionnaires, observations by the therapist, interviews, statistical findings, clinical assessment, etc.), and involve a rigorous data triangulation process to assess whether the data from different sources converge (McLeod, 2010). Since systematic case studies are methodologically pluralistic, they have a greater interest in situating patients within the study of a broader population than clinical case studies (Iwakabe & Gazzola, 2009). Systematic case studies are considered to be an accessible method for developing research evidence-base in psychotherapy (Widdowson, 2011), especially since they correct some of the methodological limitations (e.g. lack of ‘third party’ perspectives and bias in data analysis) inherent to classic clinical case studies (Iwakabe & Gazzola, 2009). They have been used for the purposes of clinical training (Tuckett, 2008), outcome assessment (Hilliard, 1993), development of clinical techniques (Almond, 2004) and meta-analysis of qualitative findings (Timulak, 2009). All these developments signal a revived interest in the case study method, but also point to the obvious lack of a research assessment tool suitable for case studies in psychotherapy (Table 1).

**Table 1:** Key concept: systematic case study

Systematic case study is a systematised alternative to the classical clinical case study. Systematic case studies generally involve a team of researchers, gather data from multiple different sources (questionnaires, observations by the therapist, interviews, statistical findings, etc.) and feature data triangulation processes in order to assess whether the data from different sources converge.	

To attend to this research gap, this paper first reviews issues around the conceptualisation of validity within the paradigms of evidence-based practice (EBP) and practice-based evidence (PBE). Although case studies are often positioned at the low end of EBP (Aveline, 2005), the paper suggests that systematic cases are a valuable form of evidence, capable of complimenting large-scale studies such as randomised controlled trials (RCTs). However, there remains a difficulty in assessing the quality and relevance of case study findings to broader psychotherapy research.

As a way forward, the paper introduces a novel Case Study Evaluation-tool (CaSE) in the form of *CaSE Purpose*-*based Evaluative Framework for Systematic Case Studies* and *CaSE Checklist for Essential Components in Systematic Case Studies*. The long-term development of CaSE would contribute to psychotherapy research and practice in three ways.

Given the significance of methodological pluralism and diverse research aims in systematic case studies, CaSE will not seek to prescribe explicit case study writing guidelines, which has already been done by numerous authors (McLeod, 2010; Meganck, Inslegers, Krivzov, & Notaerts, 2017; Willemsen et al., 2017). Instead, CaSE will enable the *retrospective* assessment of systematic case study findings and their relevance (or lack thereof) to broader psychotherapy research and practice. However, there is no reason to assume that CaSE cannot be used *prospectively* (i.e. producing systematic case studies in accordance to CaSE evaluative framework, as per point 3 in Table 2).

**Table 2:** How can Case Study Evaluation-tool (CaSE) be used in psychotherapy research and practice?

1. Using CaSE for the assessment of specific systematic case studies and their relevance to the broader field of psychotherapy research and practice;	
2. Using CaSE to evaluate the varying evidential quality of systematic case studies, which is particularly problematic for qualitative meta-analysis and meta-synthesis of published case studies in psychotherapy (Duncan & Sparks, 2020; Iwakabe & Gazzola, 2009; Thorne, Jensen, Kearney, Noblit, & Sandelowski, 2004);	
3. Using CaSE to improve the evidential quality, formulation and implications of systematic case studies in psychotherapy.	

The development of a research assessment or appraisal tool is a lengthy, ongoing process (Long & Godfrey, 2004). It is particularly challenging to develop a comprehensive *purpose*-*oriented* evaluative framework, suitable for the assessment of diverse methodologies, aims and outcomes. As such, this paper should be treated as an introduction to the broader development of CaSE tool. It will introduce the rationale behind CaSE and lay out its main approach to evidence and evaluation, with further development in mind. A case example from the Single Case Archive (SCA) (https://singlecasearchive.com) will be used to demonstrate the application of the tool ‘in action’. The paper notes further research trajectories and discusses some of the limitations around the use of the tool.

## Separating the wheat from the chaff: what is and is not evidence in psychotherapy (and who gets to decide?)

### The common approach: evidence-based practice

In the last two decades, psychotherapy has become increasingly centred around the idea of an evidence-based practice (EBP). Initially introduced in medicine, EBP has been defined as ‘conscientious, explicit and judicious use of current best evidence in making decisions about the care of individual patients’ (Sackett, Rosenberg, Gray, Haynes, & Richardson, 1996). EBP revolves around *efficacy* research: it seeks to examine whether a specific intervention has a causal (in this case, measurable) effect on clinical populations (Barkham & Mellor-Clark, 2003). From a conceptual standpoint, Sackett and colleagues defined EBP as a paradigm that is inclusive of many methodologies, so long as they contribute towards clinical decision-making process and accumulation of best currently available evidence in any given set of circumstances (Gabbay & le May, 2011). Similarly, the American Psychological Association (APA, 2010) has recently issued calls for evidence-based systematic case studies in order to produce standardised measures for evaluating process and outcome data across different therapeutic modalities.

However, given EBP’s focus on establishing cause-and-effect relationships (Rosqvist, Thomas, & Truax, 2011), it is unsurprising that qualitative research is generally not considered to be ‘gold standard’ or ‘efficacious’ within this paradigm (Aveline, 2005; Cartwright & Hardie, 2012; Edwards, 2013; Edwards, Dattilio, & Bromley, 2004; Longhofer, Floersch, & Hartmann, 2017). Qualitative methods like systematic case studies maintain an appreciation for context, complexity and meaning making. Therefore, instead of measuring regularly occurring causal relations (as in quantitative studies), the focus is on studying complex social phenomena (e.g. relationships, events, experiences, feelings, etc.) (Erickson, 2012; Maxwell, 2004). Edwards (2013) points out that, although context-based research in systematic case studies is the bedrock of psychotherapy theory and practice, it has also become shrouded by an unfortunate ideological description: ‘anecdotal’ case studies (i.e. unscientific narratives lacking evidence, as opposed to ‘gold standard’ evidence, a term often used to describe the RCT method and the therapeutic modalities supported by it), leading to a further need for advocacy in and defence of the unique epistemic process involved in case study research (Fishman, Messer, Edwards, & Dattilio, 2017).

The EBP paradigm prioritises the quantitative approach to causality, most notably through its focus on high generalisability and the ability to deal with bias through randomisation process. These conditions are associated with randomised controlled trials (RCTs) but are limited (or, as some argue, impossible) in qualitative research methods such as the case study (Margison et al., 2000) (Table 3).

**Table 3:** Key concept: evidence-based practice (EBP)

Evidence–based practice (EBP) was introduced in medicine as a conscientious use of current best evidence in clinical-decision making about individual patients. EBP revolves around efficacy research, which assesses whether specific interventions produce causal (measurable) effects on clinical populations. Internal validity and randomisation of samples are crucial to efficacy research. An example of such research is randomised controlled trials (RCTs).	

**Fig. 1:**
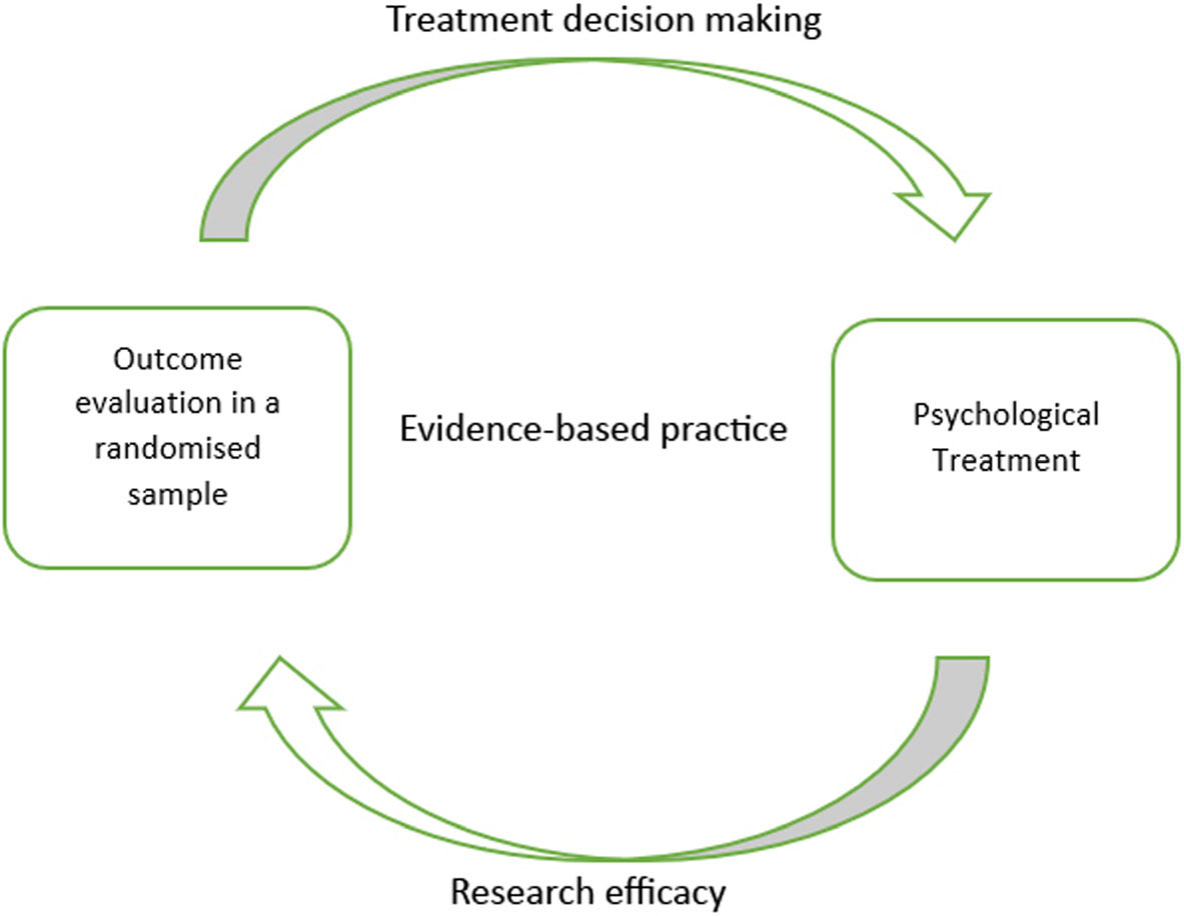
Typical knowledge generation process in evidence–based practice (EBP)

‘Evidence’ from an EBP standpoint hovers over the epistemological assumption of *procedural objectivity*: knowledge *can* be generated in a standardised, non-erroneous way, thus producing objective (i.e. with minimised bias) data. This can be achieved by anyone, as long as they are able to perform the methodological procedure (e.g. RCT) appropriately, in a ‘clearly defined and accepted process that assists with knowledge production’ (Douglas, 2004, p. 131). If there is a well-outlined quantitative form for knowledge production, the same outcome should be achieved regardless of who processes or interprets the information. For example, researchers using Cochrane Review assess the strength of evidence using meticulously controlled and scrupulous techniques; in turn, this minimises individual judgment and creates unanimity of outcomes across different groups of people (Gabbay & le May, 2011). The typical process of knowledge generation (through employing RCTs and procedural objectivity) in EBP is demonstrated in Fig. 1.

**Fig. 2:**
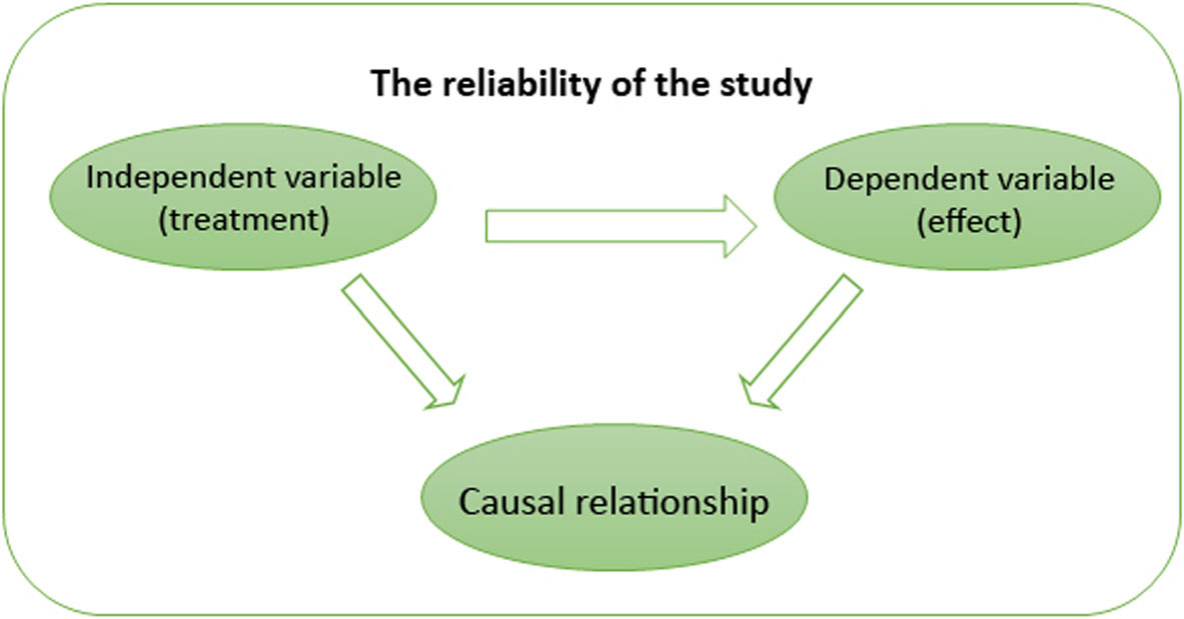
Internal validity

In EBP, the concept of validity remains somewhat controversial, with many critics stating that it limits rather than strengthens knowledge generation (Berg, 2019; Berg & Slaattelid, 2017; Lilienfeld, Ritschel, Lynn, Cautin, & Latzman, 2013). This is because efficacy research relies on *internal validity*. At a general level, this concept refers to the congruence between the research study and the research findings (i.e. the research findings were not influenced by anything *external* to the study, such as confounding variables, methodological errors and bias); at a more specific level, internal validity determines the extent to which a study establishes a reliable causal relationship between an independent variable (e.g. treatment) and independent variable (outcome or effect) (Margison et al., 2000). This approach to validity is demonstrated in Fig. 2.

Social scientists have argued that there is a trade-off between research rigour and generalisability: the more specific the sample and the more rigorously defined the intervention, the outcome is likely to be less applicable to everyday, routine practice. As such, there remains a tension between employing procedural objectivity which increases the rigour of research outcomes and applying such outcomes to routine psychotherapy practice where scientific standards of evidence are not uniform.

According to McLeod (2002), inability to address questions that are most relevant for practitioners contributed to a deepening research–practice divide in psychotherapy. Studies investigating how practitioners make clinical decisions and the kinds of evidence they refer to show that there is a strong preference for knowledge that is *not* generated procedurally, i.e. knowledge that encompasses concrete clinical situations, experiences and techniques. A study by Stewart and Chambless (2007) sought to assess how a larger population of clinicians (under APA, from varying clinical schools of thought and independent practices, sample size 591) make treatment decisions in private practice. The study found that large-scale statistical data was not the primary source of information sought by clinicians. The most important influences were identified as past clinical experiences and clinical expertise (*M* = 5.62). Treatment materials based on clinical case observations and theory (*M* = 4.72) were used almost as frequently as psychotherapy outcome research findings (*M* = 4.80) (i.e. evidence-based research). These numbers are likely to fluctuate across different forms of psychotherapy; however, they are indicative of the need for research about routine clinical settings that does not isolate or generalise the effect of an intervention but examines the variations in psychotherapy processes.

### The alternative approach: practice-based evidence

In an attempt to dissolve or lessen the research–practice divide, an alternative paradigm of practice-based evidence (PBE) has been suggested (Barkham & Mellor-Clark, 2003; Fox, 2003; Green & Latchford, 2012; Iwakabe & Gazzola, 2009; Laska, Motulsky, Wertz, Morrow, & Ponterotto, 2014; Margison et al., 2000). PBE represents a shift in how we think about evidence and knowledge generation in psychotherapy. PBE treats research as a local and contingent process (at least initially), which means it focuses on variations (e.g. in patient symptoms) and complexities (e.g. of clinical setting) in the studied phenomena (Fox, 2003). Moreover, research and theory-building are seen as complementary rather than detached activities from clinical practice. That is to say, PBE seeks to examine how and which treatments can be improved in everyday clinical practice by flagging up clinically salient issues and developing clinical techniques (Barkham & Mellor-Clark, 2003). For this reason, PBE is concerned with the *effectiveness* of research findings: it evaluates how well interventions work in real-world settings (Rosqvist et al., 2011). Therefore, although it is not unlikely for RCTs to be used in order to generate practice-informed evidence (Horn & Gassaway, 2007), qualitative methods like the systematic case study are seen as ideal for demonstrating the effectiveness of therapeutic interventions with individual patients (van Hennik, 2020) (Table 4).

**Table 4:** Key concept: practice-based evidence (PBE)

Practice-based evidence (PBE) was introduced as an alternative paradigm to EBP. PBE focuses on assessing the variations and complexities of treatment in routine clinical practice. Research in PBE is concerned with the effectiveness of findings by examining how interventions work in real-world settings. External validity and contingency of findings is crucial to effectiveness research. An example of such research is the systematic case study.	

PBE’s epistemological approach to ‘evidence’ may be understood through the process of *concordant objectivity* (Douglas, 2004): ‘Instead of seeking to eliminate individual judgment, … [concordant objectivity] checks to see whether the individual judgments of people in fact do agree’ (p. 462). This does not mean that anyone can contribute to the evaluation process like in procedural objectivity, where the main criterion is following a set quantitative protocol or knowing how to operate a specific research design. Concordant objectivity requires that there is a set of competent observers who are closely familiar with the studied phenomenon (e.g. researchers and practitioners who are familiar with depression from a variety of therapeutic approaches).

**Fig. 3:**
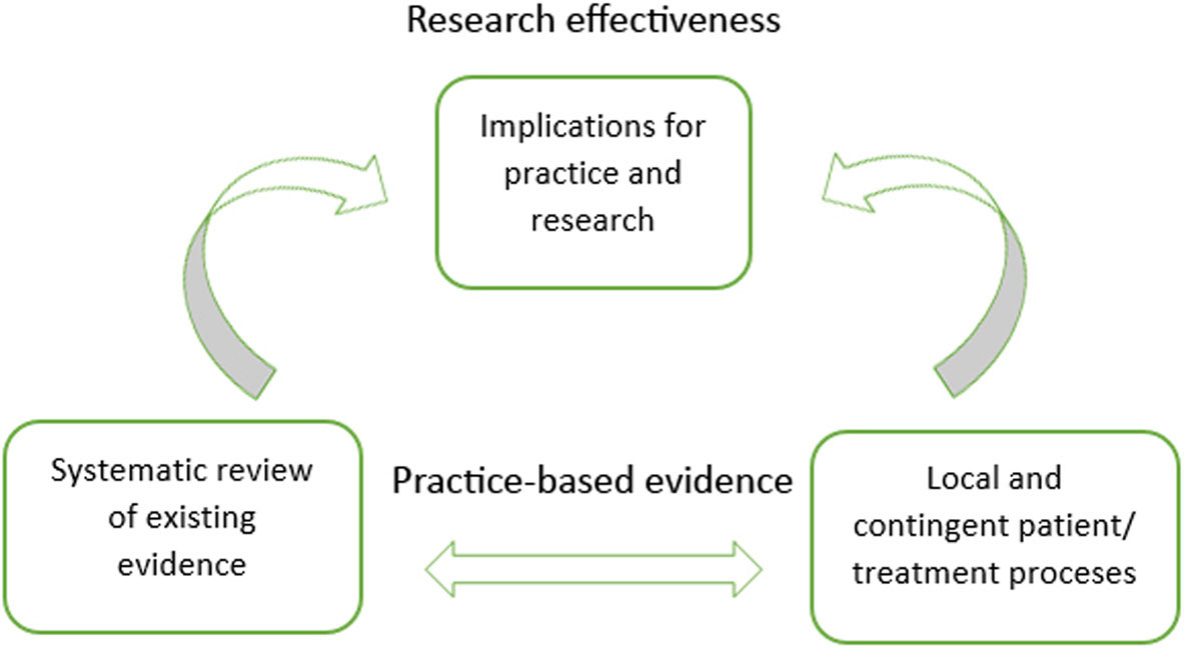
Typical knowledge generation process in practice-based evidence (PBE)

Systematic case studies are a good example of PBE ‘in action’: they allow for the examination of detailed unfolding of events in psychotherapy practice, making it the most pragmatic and practice-oriented form of psychotherapy research (Fishman, 1999, 2005). Furthermore, systematic case studies approach evidence and results through concordant objectivity (Douglas, 2004) by involving a team of researchers and rigorous data triangulation processes (McLeod, 2010). This means that, although systematic case studies remain focused on particular clinical situations and detailed subjective experiences (similar to classic clinical case studies; see Iwakabe & Gazzola, 2009), they still involve a series of validity checks and considerations on how findings from a single systematic case pertain to broader psychotherapy research (Fishman, 2005). The typical process of knowledge generation (through employing systematic case studies and concordant objectivity) in PBE is demonstrated in Fig. 3. The figure exemplifies a bidirectional approach to research and practice, which includes the development of research-supported psychological treatments (through systematic reviews of existing evidence) *as well as* the perspectives of clinical practitioners in the research process (through the study of local and contingent patient and/or treatment processes) (Teachman et al., 2012; Westen, Novotny, & Thompson-Brenner, 2004).

**Fig. 4:**
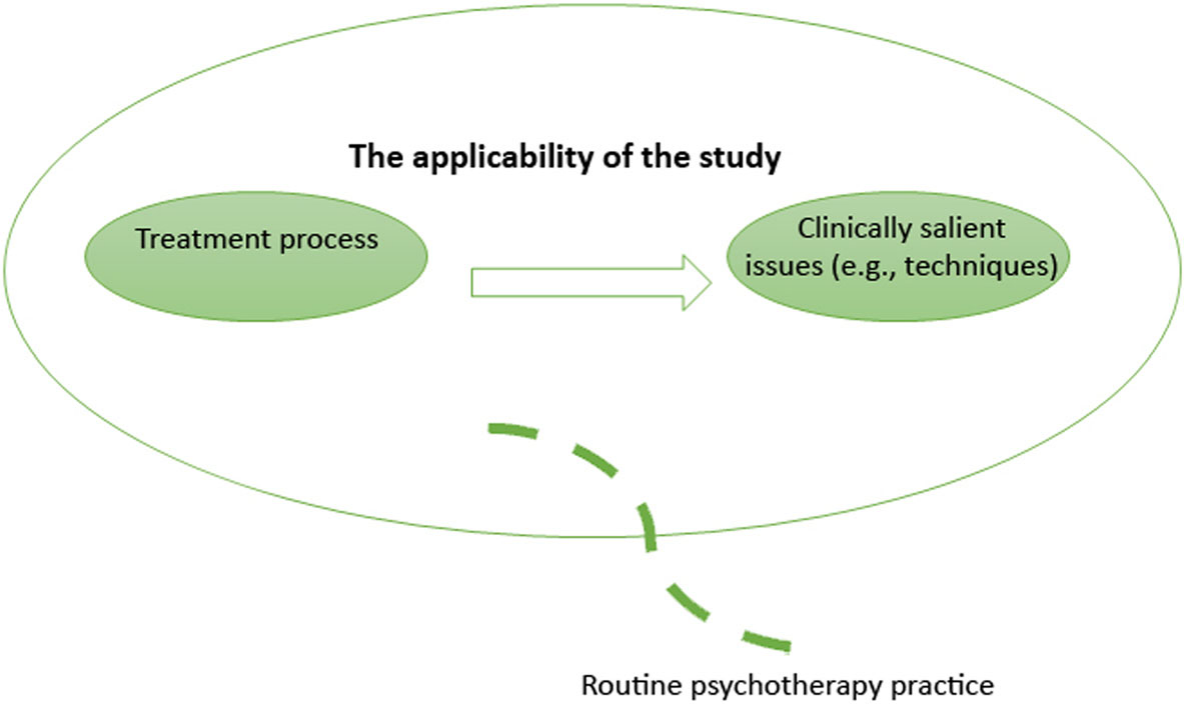
External validity

From a PBE standpoint, *external validity* is a desirable research condition: it measures extent to which the impact of interventions apply to real patients and therapists in everyday clinical settings. As such, external validity is not based on the strength of causal relationships between treatment interventions and outcomes (as in internal validity); instead, the use of specific therapeutic techniques and problem-solving decisions are considered to be important for generalising findings onto routine clinical practice (even if the findings are explicated from a single case study; see Aveline, 2005). This approach to validity is demonstrated in Fig. 4.

Since effectiveness research is less focused on limiting the context of the studied phenomenon (indeed, explicating the context is often one of the research aims), there is more potential for confounding factors (e.g. bias and uncontrolled variables) which in turn can reduce the study’s internal validity (Barkham & Mellor-Clark, 2003). This is also an important challenge for research appraisal. Douglas (2004) argues that appraising research in terms of its effectiveness may produce significant disagreements or group illusions, since what might work for some practitioners may not work for others: ‘It cannot guarantee that values are not influencing or supplanting reasoning; the observers may have shared values that cause them to all disregard important aspects of an event’ (Douglas, 2004, p. 462). Douglas further proposes that an *interactive approach to objectivity* may be employed as a more complex process in debating the evidential quality of a research study: it requires a discussion among observers and evaluators in the form of peer-review, scientific discourse, as well as research appraisal tools and instruments. While these processes of rigour are also applied in EBP, there appears to be much more space for debate, disagreement and interpretation in PBE’s approach to research evaluation, partly because the evaluation criteria themselves are subject of methodological debate and are often employed in different ways by researchers (Williams et al., 2019). This issue will be addressed more explicitly again in relation to CaSE development (‘Developing purpose-oriented evaluation criteria for systematic case studies’ section).

### A third way approach to validity and evidence

The research–practice divide shows us that there may be something significant in establishing complementarity between EBP and PBE rather than treating them as mutually exclusive forms of research (Fishman et al., 2017). For one, EBP is not a sufficient condition for delivering research relevant to practice settings (Bower, 2003). While RCTs can demonstrate that an intervention works on average in a group, clinicians who are facing individual patients need to answer a different question: *how can I make therapy work with this particular case*? (Cartwright & Hardie, 2012). Systematic case studies are ideal for filling this gap: they contain descriptions of microprocesses (e.g. patient symptoms, therapeutic relationships, therapist attitudes) in psychotherapy practice that are often overlooked in large-scale RCTs (Iwakabe & Gazzola, 2009). In particular, systematic case studies describing the use of specific interventions with less researched psychological conditions (e.g. childhood depression or complex post-traumatic stress disorder) can deepen practitioners’ understanding of effective clinical techniques before the results of large-scale outcome studies are disseminated.

Secondly, establishing a working relationship between systematic case studies and RCTs will contribute towards a more pragmatic understanding of validity in psychotherapy research. Indeed, the very tension and so-called trade-off between internal and external validity is based on the assumption that research methods are designed on an either/or basis; either they provide a sufficiently rigorous study design or they produce findings that can be applied to real-life practice. Jimenez-Buedo and Miller (2010) call this assumption into question: in their view, if a study is not internally valid, then ‘little, or rather nothing, can be said of the outside world’ (p. 302). In this sense, internal validity may be seen as a pre-requisite for any form of applied research and its external validity, but it need not be constrained to the quantitative approach of causality. For example, Levitt, Motulsky, Wertz, Morrow, and Ponterotto (2017) argue that, what is typically conceptualised as internal validity, is, in fact, a much broader construct, involving the assessment of how the research method (whether qualitative or quantitative) is best suited for the research goal, and whether it obtains the relevant conclusions. Similarly, Truijens, Cornelis, Desmet, and De Smet (2019) suggest that we should think about validity in a broader epistemic sense—not just in terms of psychometric measures, but also in terms of the research design, procedure, goals (research questions), approaches to inquiry (paradigms, epistemological assumptions), etc.

The overarching argument from research cited above is that all forms of research—qualitative and quantitative—can produce ‘valid evidence’ but the validity itself needs to be assessed against each specific research method and purpose. For example, RCTs are accompanied with a variety of clearly outlined appraisal tools and instruments such as CASP (Critical Appraisal Skills Programme) that are well suited for the assessment of RCT validity and their implications for EBP. Systematic case studies (or case studies more generally) currently have no appraisal tools in any discipline. The next section evaluates whether existing qualitative research appraisal tools are relevant for systematic case studies in psychotherapy and specifies the missing evaluative criteria.

## The relevance of existing appraisal tools for qualitative research to systematic case studies in psychotherapy

### What is a research tool?

Currently, there are several research appraisal tools, checklists and frameworks for qualitative studies. It is important to note that tools, checklists and frameworks are not equivalent to one another but actually refer to different approaches to appraising the validity of a research study. As such, it is erroneous to assume that all forms of qualitative appraisal feature the same aims and methods (Hannes et al., 2010; Williams et al., 2019).

Generally, research assessment falls into two categories: *checklists* and *frameworks*. Checklist approaches are often contrasted with quantitative research, since the focus is on assessing the internal validity of research (i.e. researcher’s independence from the study). This involves the assessment of bias in sampling, participant recruitment, data collection and analysis. Framework approaches to research appraisal, on the other hand, revolve around traditional qualitative concepts such as transparency, reflexivity, dependability and transferability (Williams et al., 2019). Framework approaches to appraisal are often challenging to use because they depend on the reviewer’s familiarisation and interpretation of the qualitative concepts.

Because of these different approaches, there is some ambiguity in terminology, particularly between *research appraisal instruments* and *research appraisal tools*. These terms are often used interchangeably in appraisal literature (Williams et al., 2019). In this paper, research appraisal tool is defined as a method-specific (i.e. it identifies a specific research method or component) form of appraisal that draws from both checklist and framework approaches. Furthermore, a research appraisal tool seeks to inform decision making in EBP or PBE paradigms and provides explicit definitions of the tool’s evaluative framework (thus minimising—but by no means eliminating—the reviewers’ interpretation of the tool). This definition will be applied to CaSE (Table 5).

**Table 5:** Key concept: research appraisal tool

Research appraisal tool is a method-specific (or a research component-specific) form of appraisal that draws from both checklist and framework approaches. A research appraisal tool will usually provide explicit definitions for its evaluative framework and will be used by researchers who wish to demonstrate the evidential quality of their study to the readers.	

In contrast, research appraisal instruments are generally seen as a broader form of appraisal in the sense that they may evaluate a variety of methods (i.e. they are non-method specific or they do not target a particular research component), and are aimed at checking whether the research findings and/or the study design contain specific elements (e.g. the aims of research, the rationale behind design methodology, participant recruitment strategies, etc.).

There is often an implicit difference in audience between appraisal tools and instruments. Research appraisal instruments are often aimed at researchers who want to assess the strength of their study; however, the process of appraisal may not be made explicit in the study itself (besides mentioning that the tool was used to appraise the study). Research appraisal tools are aimed at researchers who wish to explicitly demonstrate the evidential quality of the study to the readers (which is particularly common in RCTs). All forms of appraisal used in the comparative exercise below are defined as ‘tools’, even though they have different appraisal approaches and aims.

### Comparing different qualitative tools

Hannes et al. (2010) identified CASP (Critical Appraisal Skills Programme-tool), JBI (Joanna Briggs Institute-tool) and ETQS (Evaluation Tool for Qualitative Studies) as the most frequently used critical appraisal tools by qualitative researchers. All three instruments are available online and are free of charge, which means that any researcher or reviewer can readily utilise CASP, JBI or ETQS evaluative frameworks to their research. Furthermore, all three instruments were developed within the context of organisational, institutional or consortium support (Tables 6, 7 and 8).

**Table 6:** CASP (Critical Appraisal Skills Programme-tool)

CASP is part of the Oxford Centre for Triple Value Healthcare enterprise, which seeks to support healthcare systems and achieve optimal outcomes for populations. CASP has a variety of checklists, many of which are aimed at RCTs (e.g. RCT checklist, systematic review checklist, cohort study checklist, etc.).

**Table 7:** JBI (Joanna Briggs Institute-tool)

JBI was developed by the Joanna Briggs Institute led by Alan Pearson. Like CASP, JBI offers a variety of appraisal checklists (e.g. cross sectional studies, diagnostic test accuracy studies, cohort studies, etc.) that are aimed at improving healthcare research and practice.

**Table 8:** ETQS (Evaluation Tool for Qualitative Studies)

ETQS was developed at the University of Leeds by Andrew Long in the Department of Health, under the Outcomes for Social Care for Adults (OSCA) Initiative (1997–1999). Out of the three tools, ETQS is most attuned to the qualitative research paradigm; it seeks to assess and enhance evidence that is ‘different’ from the common efficacy research in EBP (Long & Godfrey, 2004).	

It is important to note that neither of the three tools is specific to systematic case studies or psychotherapy case studies (which would include not only systematic but also experimental and clinical cases). This means that using CASP, JBI or ETQS for case study appraisal may come at a cost of overlooking elements and components specific to the systematic case study method.

Based on Hannes et al. (2010) comparative study of qualitative appraisal tools as well as the different evaluation criteria explicated in CASP, JBI and ETQS evaluative frameworks, I assessed how well each of the three tools is attuned to the *methodological*, *clinical* and *theoretical* aspects of systematic case studies in psychotherapy. The latter components were based on case study guidelines featured in the journal of *Pragmatic Case Studies in Psychotherapy* as well as components commonly used by published systematic case studies across a variety of other psychotherapy journals (e.g. *Psychotherapy Research*, *Research In Psychotherapy*: *Psychopathology Process And Outcome*, etc.) (see Table 9 for detailed descriptions of each component).

**Table 9:** Comparing the relevance of JBI (Joanna Briggs Institute), CASP (Critical Appraisal Skills Program) and ETQS (Evaluation Tool for Qualitative Studies) for appraising components specific to systematic case studies

Systematic case study components	JBI Evaluation Criteria	CASP Evaluation Criteria	ETQS Evaluation Criteria
Methodological components			
**Case context and method**	Congruity between the research methodology and the research question or objectives	Methodological screening questions: Is a qualitative methodology appropriate? Was the research design appropriate to address the aims of the research?	No assessment criteria for the suitability of the case study method
**Research participants (description of patients, therapists, researchers)**	Cultural and theoretical context of the researcher; researcher’s impact on the research (and vice versa); adequate patient representation	No assessment criteria for the description of researchers and data analysts	How do the authors locate the study within the existing knowledge base? What role does the researcher adopt within the setting? Are the researcher’s own position, assumptions, and possible biases outlined?
**Research procedure (data collection and analysis methods)**	Congruity between the research methodology and the analysis of data and interpretation of results	Was the recruitment strategy appropriate to the aims of the research? Were the data collected in a way that addressed the research issue? Was the data analysis sufficiently rigorous?	What theoretical framework guides or informs the study? What data collection methods are used to obtain and record data? How were data analysed?
Clinical components			
**Case introduction**	Clear description of patient demographics and current clinical condition	No assessment criteria for case description	What are the key characteristics of the sample (events, persons, times and settings)?
**Assessment of the client’s problems, goals, strengths, and history (includes many data sources and methods, such as diagnostic tools and questionnaires)**	Participants and their voices are clearly represented No assessment criteria for patient’s clinical assessment or the use of other methods and data sources No assessment criteria for the formulation and planning of the treatment	No assessment criteria for patient’s clinical assessment or the use of other methods and data sources	Within what geographical and care setting is the study carried out? Is sufficient detail given about the setting? No assessment criteria for patient’s clinical assessment or the use of other methods and data sources
**Course of therapy and treatment plan**	Clear description of patient’s history, including a timeline of relevant events	No assessment criteria for course of treatment or progress	Over what time period is the study conducted? No assessment criteria for therapeutic progress
Theoretical components			
**Clinical decision-making (includes assessment of clinical outcomes and theoretical findings)**	Research conclusions flow from the analysis and interpretation of the data	Is there a clear statement of findings? (e.g. triangulation, respondent validation, more than one analyst)	Is there sufficient breadth (e.g. contrast of two or more perspective) and depth (e.g. insight into a single perspective)? What are the implications for policy and practice?
**Research limitations**	No assessment criteria for research limitations	No assessment criteria for research limitations	Is there evidence of reflexivity? Is adequate evidence provided to support the analysis (validity and reliability)?
**Transferability of findings**	No assessment criteria for transferability of findings	How valuable is the research? Consider the findings in relation to current practice or policy, or relevant research-based literature and how findings can be transferred to other populations or other ways in which the research may be used	To what setting and population are the study findings generalizable?

The evaluation criteria for each tool in Table 9 follows Joanna Briggs Institute (JBI) (2017a, 2017b); Critical Appraisal Skills Programme (CASP) (2018); and *ETQS Questionnaire* (first published in 2004 but revised continuously since). Table 10 demonstrates how each tool should be used (i.e. recommended reviewer responses to checklists and questionnaires).

**Table 10:** Recommended reviewer responses to JBI (Joanna Briggs Institute), CASP (Critical Appraisal Skills Program) and ETQS (Evaluation Tool for Qualitative Studies)

JBI evaluation responses	CASP evaluation responses	ETQS evaluation responses
Checklist: Yes No Unclear Not applicable	Checklist: Yes No Can’t tell Additional space for comments available	Open–ended questionnaire: Comprehensive and detailed responses in relation to the study

### Using CASP, JBI and ETQS for systematic case study appraisal

Although JBI, CASP and ETQS were all developed to appraise qualitative research, it is evident from the above comparison that there are significant differences between the three tools. For example, JBI and ETQS are well suited to assess researcher’s interpretations (Hannes et al. (2010) defined this as *interpretive validity*, a subcategory of *internal validity*): the researcher’s ability to portray, understand and reflect on the research participants’ experiences, thoughts, viewpoints and intentions. JBI has an explicit requirement for participant voices to be clearly represented, whereas ETQS involves a set of questions about key characteristics of events, persons, times and settings that are relevant to the study. Furthermore, both JBI and ETQS seek to assess the researcher’s influence on the research, with ETQS particularly focusing on the evaluation of *reflexivity* (the researcher’s personal influence on the interpretation and collection of data). These elements are absent or addressed to a lesser extent in the CASP tool.

The appraisal of transferability of findings (what this paper previously referred to as *external validity*) is addressed only by ETQS and CASP. Both tools have detailed questions about the value of research to practice and policy as well as its transferability to other populations and settings. Methodological research aspects are also extensively addressed by CASP and ETQS, but less so by JBI (which relies predominantly on congruity between research methodology and objectives without any particular assessment criteria for other data sources and/or data collection methods). Finally, the evaluation of theoretical aspects (referred to by Hannes et al. (2010) as *theoretical validity*) is addressed only by JBI and ETQS; there are no assessment criteria for theoretical framework in CASP.

Given these differences, it is unsurprising that CASP, JBI and ETQS have limited relevance for systematic case studies in psychotherapy. First, it is evident that neither of the three tools has specific evaluative criteria for the clinical component of systematic case studies. Although JBI and ETQS feature some relevant questions about *participants* and their context, the conceptualisation of *patients* (and/or clients) in psychotherapy involves other kinds of data elements (e.g. diagnostic tools and questionnaires as well as therapist observations) that go beyond the usual participant data. Furthermore, much of the clinical data is intertwined with the therapist’s clinical decision-making and thinking style (Kaluzeviciute & Willemsen, 2020). As such, there is a need to appraise patient data and therapist interpretations not only on a separate basis, but also as two forms of knowledge that are deeply intertwined in the case narrative.

Secondly, since systematic case studies involve various forms of data, there is a need to appraise how these data converge (or how different methods complement one another in the case context) and how they can be transferred or applied in broader psychotherapy research and practice. These systematic case study components are attended to a degree by CASP (which is particularly attentive of methodological components) and ETQS (particularly specific criteria for research transferability onto policy and practice). These components are not addressed or less explicitly addressed by JBI. Overall, neither of the tools is attuned to all methodological, theoretical and clinical components of the systematic case study. Specifically, there are no clear evaluation criteria for the description of research teams (i.e. different data analysts and/or clinicians); the suitability of the systematic case study method; the description of patient’s clinical assessment; the use of other methods or data sources; the general data about therapeutic progress.

Finally, there is something to be said about the recommended reviewer responses (Table 10). Systematic case studies can vary significantly in their formulation and purpose. The methodological, theoretical and clinical components outlined in Table 9 follow guidelines made by case study journals; however, these are recommendations, not ‘set in stone’ case templates. For this reason, the straightforward checklist approaches adopted by JBI and CASP may be difficult to use for case study researchers and those reviewing case study research. The ETQS open-ended questionnaire approach suggested by Long and Godfrey (2004) enables a comprehensive, detailed and purpose-oriented assessment, suitable for the evaluation of systematic case studies. That said, there remains a challenge of ensuring that there is less space for the interpretation of evaluative criteria (Williams et al., 2019). The combination of checklist and framework approaches would, therefore, provide a more stable appraisal process across different reviewers.

## Developing purpose-oriented evaluation criteria for systematic case studies

The starting point in developing evaluation criteria for Case Study Evaluation-tool (CaSE) is addressing the significance of pluralism in systematic case studies. Unlike RCTs, systematic case studies are pluralistic in the sense that they employ divergent practices in methodological procedures (*research process*), and they may include significantly different research aims and purpose (*the end*-*goal*) (Kaluzeviciute & Willemsen, 2020). While some systematic case studies will have an explicit intention to conceptualise and situate a single patient’s experiences and symptoms within a broader clinical population, others will focus on the exploration of phenomena as they emerge from the data. It is therefore important that CaSE is positioned within a *purpose*-*oriented evaluative framework*, suitable for the assessment of what each systematic case is good for (rather than determining an absolute measure of ‘good’ and ‘bad’ systematic case studies). This approach to evidence and appraisal is in line with the PBE paradigm. PBE emphasises the study of clinical complexities and variations through local and contingent settings (e.g. single case studies) and promotes methodological pluralism (Barkham & Mellor-Clark, 2003).

### CaSE checklist for essential components in systematic case studies

In order to conceptualise purpose-oriented appraisal questions, we must first look at what unites and differentiates systematic case studies in psychotherapy. The commonly used theoretical, clinical and methodological systematic case study components were identified earlier in Table 9. These components will be seen as *essential* and common to most systematic case studies in CaSE evaluative criteria. If these essential components are missing in a systematic case study, then it may be implied there is a lack of information, which in turn diminishes the evidential quality of the case. As such, the checklist serves as a tool for checking whether a case study is, indeed, systematic (as opposed to experimental or clinical; see Iwakabe & Gazzola, 2009 for further differentiation between methodologically distinct case study types) and should be used before *CaSE Purpose*-*based Evaluative Framework for Systematic Case Studie*s (which is designed for the appraisal of different purposes common to systematic case studies).

As noted earlier in the paper, checklist approaches to appraisal are useful when evaluating the presence or absence of specific information in a research study. This approach can be used to appraise essential components in systematic case studies, as shown below. From a pragmatic point view (Levitt et al., 2017; Truijens et al., 2019), *CaSE Checklist for Essential Components in Systematic Case Studies* can be seen as a way to ensure the internal validity of systematic case study: the reviewer is assessing whether sufficient information is provided about the case design, procedure, approaches to inquiry, etc., and whether they are relevant to the researcher’s objectives and conclusions (Table 11).

**Table 11:** Case Study Evaluation-tool (CaSE) checklist for essential components in systematic case studies. Recommended responses: Yes, No, unclear or not applicable

**Methodology**	
1. The rationale behind choosing the case study method	
2. Description of research design and aims	
3. Description of research participants, including:	
3a. Patients/clients	
3b. Therapists, clinical supervisors	
3c. Researchers/data analysts (research team)	
4. Description of research procedures, including:	
4a. Evaluation of existing literature and research	
4b. Data collection methods	
4c. Data analysis methods	
4d. Data triangulation procedures	
4e. Research appraisal tools and instruments	
5. Description of researchers’ reflexivity (awareness of the relationship between the researcher and research study), including:	
5a. Research assumptions pertaining to objectives	
5b. Research biases pertaining to data analysis	
5c. Differentiation between assumptions and views made by different researchers/therapists	
6. Description of research limitations, including:	
6a. Congruity between research data and research aims and objectives	
6b. Research appraisal and validity	
7. Relevant ethical information, including:	
7a. Patient’s informed consent	
7b. Anonymisation of specific clinical material	
**Clinical components**	
8. Description of patient’s history, including:	
8a. Demographics	
8b. Cultural context	
8c. Socio-economic context	
8d. Interpersonal history (family and other relationships)	
9. Description of patient’s clinical condition, including:	
9a. Current and past diagnosis (with reference to DSM, ICD and other diagnostic manuals)	
9b. Current and past symptoms and experiences	
9c. Previously received treatment	
9d. The use of medication	
10. Description of patient’s problems through:	
10a. Diagnostic tools (therapist’s assessment)	
10b. Self–report questionnaires (patient’s self–assessment)	
11. Description of course of therapy and treatment, including:	
11a. Therapeutic modality	
11b. Therapeutic setting (number of sessions, frequency, private/public practice)	
11c. Therapeutic relationship	
11d. Timeline of relevant treatment events/sessions	
11e. Follow-up information	
11f. Treatment outcomes	
11g. Complicating factors	
12. Description of clinical decision–making and reflexivity (awareness of the relationship between the therapist and the treatment process), including:	
12a. Clinical assumptions pertaining to diagnosis	
12b. Clinical biases pertaining to therapeutic techniques and interpretations (especially in relation to therapist’s therapeutic modality)	
13. Description of therapist where relevant, including:	
13a. Professional experience	
13b. Demographics	
13c. Cultural context	
13d. Socio-economic context	
**Theory**	
14. Clear description of theoretical references and key concepts	
15. Description of how clinical decision–making relates to the chosen theoretical framework	
16. Clear statement of theoretical findings	
17. Clear description of evidence for and limitations of the chosen theoretical framework, including:	
17a. Validity (does the case study attend its research objectives and aims sufficiently? Do researchers use relevant theoretical concepts, clinical techniques and research methods?)	
17b. Reliability (does the case study provide sufficient, detailed and reflexive information on how it arrived at its findings?)	
18. Description of transferability of findings (relevance to other cases), including:	
18a. Transferability to psychotherapy research	
18b. Transferability to psychotherapy practice	
18c. Relevance to policy in private and/or public healthcare	
18d. Relevance to specific clinical population and setting	

### CaSE purpose-based evaluative framework for systematic case studies

Identifying differences between systematic case studies means identifying the different purposes systematic case studies have in psychotherapy. Based on the earlier work by social scientist Yin (1984, 1993), we can differentiate between *exploratory* (hypothesis generating, indicating a beginning phase of research), *descriptive* (particularising case data as it emerges) and *representative* (a case that is typical of a broader clinical population, referred to as the ‘explanatory case’ by Yin) cases.

Another increasingly significant strand of systematic case studies is *transferable* (aggregating and transferring case study findings) cases. These cases are based on the process of meta-synthesis (Iwakabe & Gazzola, 2009): by examining processes and outcomes in many different case studies dealing with similar clinical issues, researchers can identify common themes and inferences. In this way, single case studies that have relatively little impact on clinical practice, research or health care policy (in the sense that they capture psychotherapy processes rather than produce generalisable claims as in Yin’s *representative* case studies) can contribute to the generation of a wider knowledge base in psychotherapy (Iwakabe, 2003, 2005). However, there is an ongoing issue of assessing the evidential quality of such transferable cases. According to Duncan and Sparks (2020), although meta-synthesis and meta-analysis are considered to be ‘gold standard’ for assessing interventions across disparate studies in psychotherapy, they often contain case studies with significant research limitations, inappropriate interpretations and insufficient information. It is therefore important to have a research appraisal process in place for selecting transferable case studies.

Two other types of systematic case study research include: *critical* (testing and/or confirming existing theories) cases, which are described as an excellent method for falsifying existing theoretical concepts and testing whether therapeutic interventions work in practice with concrete patients (Kaluzeviciute, 2021), and *unique* (going beyond the ‘typical’ cases and demonstrating deviations) cases (Merriam, 1998). These two systematic case study types are often seen as less valuable for psychotherapy research given that unique/falsificatory findings are difficult to generalise. But it is clear that practitioners and researchers in our field seek out context-specific data, as well as detailed information on the effectiveness of therapeutic techniques in single cases (Stiles, 2007) (Table 12).

**Table 12:** Key concept: purpose–based systematic case studies

1. **Representative cases** of a broader clinical population (*typicality*);	
2. **Descriptive cases** that capture specific psychotherapy processes as they emerge in treatment (*particularity*);	
3. **Unique cases** due to unusual variations that go beyond the ‘average’ population (*deviation*);	
4. **Critical cases** that test existing theories (*faslficiation/confirmation*);	
5. **Exploratory cases** that indicate a beginning phase of a multiple case study research (*hypothesis generation*);	
6. **Transferable cases** that seek to aggregate and transfer case study findings onto other cases (*generalisability*).	

Each purpose-based case study contributes to PBE in different ways. *Representative cases* provide qualitatively rich, in-depth data about a clinical phenomenon within its particular context. This offers other clinicians and researchers access to a ‘closed world’ (Mackrill & Iwakabe, 2013) containing a wide range of attributes about a conceptual type (e.g. clinical condition or therapeutic technique). *Descriptive cases* generally seek to demonstrate a realistic snapshot of therapeutic processes, including complex dynamics in therapeutic relationships, and instances of therapeutic failure (Maggio, Molgora, & Oasi, 2019). Data in descriptive cases should be presented in a transparent manner (e.g. if there are issues in standardising patient responses to a self-report questionnaire, this should be made explicit). Descriptive cases are commonly used in psychotherapy training and supervision. *Unique cases* are relevant for both clinicians and researchers: they often contain novel treatment approaches and/or introduce new diagnostic considerations about patients who deviate from the clinical population. *Critical cases* demonstrate the application of psychological theories ‘in action’ with particular patients; as such, they are relevant to clinicians, researchers and policymakers (Mackrill & Iwakabe, 2013). *Exploratory cases* bring new insight and observations into clinical practice and research. This is particularly useful when comparing (or introducing) different clinical approaches and techniques (Trad & Raine, 1994). Findings from exploratory cases often include future research suggestions. Finally, *transferable cases* provide one solution to the generalisation issue in psychotherapy research through the previously mentioned process of meta-synthesis. Grouped together, transferable cases can contribute to theory building and development, as well as higher levels of abstraction about a chosen area of psychotherapy research (Iwakabe & Gazzola, 2009).

With this plurality in mind, it is evident that CaSE has a challenging task of appraising research components that are *distinct* across six different types of purpose-based systematic case studies. The purpose-specific evaluative criteria in Table 13 was developed in close consultation with epistemological literature associated with each type of case study, including: Yin’s (1984, 1993) work on establishing the typicality of representative cases; Duncan and Sparks’ (2020) and Iwakabe and Gazzola’s (2009) case selection criteria for meta-synthesis and meta-analysis; Stake’s (1995, 2010) research on particularising case narratives; Merriam’s (1998) guidelines on distinctive attributes of unique case studies; Kennedy’s (1979) epistemological rules for generalising from case studies; Mahrer’s (1988) discovery oriented case study approach; and Edelson’s (1986) guidelines for rigorous hypothesis generation in case studies.

**Table 13:** Case Study Evaluation-tool (CaSE) purpose-based evaluative framework for systematic case studies. Recommended responses: open-ended questionnaire

**1 Representative cases** (purpose: *typicality*)	
**The studied phenomenon**	
• What is the studied phenomenon or ‘conceptual type’ (*e.g, clinical condition, therapeutic technique, patient’s symptoms*)? There is generally one specific phenomenon.	
• Is the studied phenomenon sufficiently distinguished from other kinds of (potentially similar) phenomena?	
**Patient data**	
• Are patient characteristics relevant to the wider clinical population? (*e.g. is there a good match between symptoms and experiences?*)	
• What is the rationale for choosing this patient?	
• Does the patient present any unique or deviant characteristics? (*e.g. symptoms that are not representative of the studied clinical condition*)	
**The clinical discourse**	
• Is there a detailed clinical narrative in the form of therapist reflections and observations?	
• Does the case move from the particularity of the patient to a more general (theoretically abstract) claim about the studied phenomenon?	
**Research**	
• Is there a sufficient review of literature on the studied phenomenon?	
• Does the case refer to other cases and/or studies that replicate their findings?	
**Case purpose**	
• Does the case demonstrate the typical characteristics of the studied phenomenon?	
• Does the case provide findings relevant for the broader clinical population?	
• Can the case contribute to psychotherapy theory?	
**2 Descriptive cases** (purpose: *particularity*)	
**The studied phenomenon**	
• What phenomena are studied in the case (*e.g. clinical condition, therapeutic technique, patient’s symptoms)*? There can be multiple phenomena.	
• Does the case present events and processes common to clinical practice? (*e.g. therapeutic relationship difficulties*)	
**Patient data**	
• Are patient characteristics described in detail, with particular attention to uniqueness, subjectivity and meaning of “lived experiences”?	
• Does the case narrative convey interpersonally sharable statements, ruminations, metaphors?	
• Is the patient clearly positioned within their cultural and psycho-social context?	
**The clinical discourse**	
• Does the case convey the process behind therapist’s practical decisions in the consulting room?	
• Does the case provide ‘know-how’ knowledge on how practitioners can deal with clinically salient issues and situations?	
• Does the therapist provide a reflexive account on how their views and theoretical assumptions might impact the therapeutic relationship and clinical decision-making?	
**Research**	
• Does the case include patient’s self-assessment? (*e.g. through self-report measures and dialogic exchange)*	
• Does the case include excerpts of dialogue between therapist and patient?	
**Case purpose**	
• Does the case provide a relational understanding (with which readers can empathise) of the studied phenomenon?	
• Does the case narrative sufficiently portray ‘real analytic practice’ rather than ‘ideal models’? (*e.g. by demonstrating disparity between clinical theory/research and practice*)	
• Can the case contribute to psychotherapy training and practice?	
**3 Unique cases** (purpose: *deviation*)	
**The studied phenomenon**	
• What phenomena are studied in the case (*e.g. clinical condition, therapeutic technique, patient’s symptoms*)? There can be multiple phenomena.	
• Does the case explain how the studied phenomena are different or unique from the established theory/research? (*e.g. the patient’s experience of transference is different from the experiences of transference across a broader clinical population*)	
**Patient data**	
• Are patient characteristics described in detail, with particular attention to uniqueness, subjectivity and meaning of “lived experiences”?	
• What is the rationale for choosing this patient?	
**The clinical discourse**	
• Does the case convey a detailed description of therapeutic interventions and their effectiveness?	
• Does the therapist provide a reflexive account on how their views and theoretical assumptions might impact clinical decision–making, particularly in terms of their understanding of the uniqueness/deviation in the case?	
• Does the case include sufficient considerations as to the cause of the deviation/uniqueness in patient’s clinical condition or symptoms?	
**Research**	
• Does the case convey more than one theoretical and/or research perspective? (*e.g. clinical assessment by multiple practitioners or data analysis by multiple researchers*)	
• Are there considerations of alternative explanations to the observed deviation/uniqueness of the case? (*e.g. by referring to other published case studies or research*)	
**Case purpose**	
• Does the case provide insight into a novel phenomenon? (e*.g. by describing unique patient symptoms or experiences*)	
• Does the case provide novel theoretical knowledge in relation to unique/deviant phenomenon? (*E.g., by developing a new therapeutic technique*)	
• Can the case contribute to psychotherapy theory, training and/or practice?	
**4 Critical cases** (purpose: *falsification/confirmation*)	
**The studied phenomenon**	
• What is the studied phenomenon in the case (*e.g. clinical condition, therapeutic technique, patient’s symptoms)*? There is generally one specific phenomenon.	
• Does the case seek to test an existing theory/research about the studied phenomenon? (*e.g. testing the effectiveness of a well–established therapeutic intervention*)	
**Patient data**	
• Are patient characteristics described in detail?	
• Is the patient clearly outlined within their cultural and psycho-social context?	
• What is the rationale for choosing this patient?	
**The clinical discourse**	
• Does the case link therapist narrative and observations with the theoretical/research considerations?	
• Does the case convey a detailed description of therapeutic interventions and their effectiveness?	
**Research**	
• Does the case convey more than one theoretical and/or research perspective? (*e.g. clinical assessment by multiple practitioners or data analysis by multiple researchers*)	
• Does the case show how the theory/research that is being tested accounts for the clinical observations in the case?	
• Does the case provide a sufficient explanation on why their chosen theory/research is more appropriate than another?	
• If the case falsifies an existing theory/research, are there sufficient sample considerations? (*e.g. the case may be unique and therefore the original theory/research still stands*)	
**Case purpose**	
• Does the case examine an existing theory/research successfully? (*e.g. by showing whether a theory is effective with a specific patient*)	
• If the case falsifies an existing theory/research, does it offer any novel suggestions or revisions to the falsified theory/research?	
• If the case confirms an existing theory/research, does it rule out alternative explanations for the tested hypothesis? (*e.g. to show that a therapeutic intervention is effective, the positive effects of other variables like medication may need to be ruled out*)	
**5 Exploratory cases** (purpose: *hypothesis generation*)	
**The studied phenomenon**	
• What phenomena are studied in the case (*e.g. clinical condition, therapeutic technique, patient’s symptoms)*? There can be multiple phenomena.	
• Is the case discovery-led, in the sense that it explores data as it emerges?	
• Does the case contain new hypotheses about the studied phenomena?	
**Patient data**	
• Are patient characteristics described in detail?	
• Is the patient clearly outlined within their cultural and psycho-social context?	
**The clinical discourse**	
• Does the case link therapist narrative and observations with the theoretical/research considerations?	
• Does the case narrative explore the ‘how’ and ‘what’ questions in relation to patient experiences and treatment processes?	
• Does the case identify complex processes and mechanisms in the treatment and link them to theory?	
**Research**	
• Is there a sufficient review of literature of the studied phenomenon?	
• Does the case convey more than one theoretical and/or research perspective? (*e.g. clinical assessment by multiple practitioners or data analysis by multiple researchers*)	
• Does the data converge? Are different/conflicting findings reported?	
**Case purpose**	
• Does the case convey more than one set of outcomes?	
• Does the case indicate future research trajectories?	
• Can the case contribute to psychotherapy theory, training and/or practice?	
**6 Transferable cases** (purpose: *generalisability*)	
**The studied phenomenon**	
• What is studied phenomenon (*e.g. clinical condition, therapeutic technique, patient’s symptoms*)? There is generally one specific phenomenon.	
• Is the studied phenomenon explicitly defined and differentiated from other kinds of (potentially similar) phenomena?	
**Patient data**	
• Are patient characteristics described in detail?	
• Is the patient clearly outlined within their cultural and psycho-social context?	
• Does the patient present characteristics typical of the studied phenomenon? Is there sufficient information (clinical, theoretical) to link the patient with the studied phenomenon?	
**The clinical discourse**	
• Is there a detailed clinical narrative in the form of therapist reflections and observations?	
• Does the case shed light on specific characteristics of the therapeutic process? (*e.g. the development of therapeutic alliance*)	
• Is the case narrative theme-focused? (*e.g. the case identifies specific treatment patterns across different sessions)*	
• Is there a clear description of the therapeutic process, usually involving a session-by-session description?	
**Research**	
• Is there a sufficient review of literature on the studied phenomenon?	
• Does the case involve a specific therapeutic, theoretical and research framework, and is the framework made explicit by the researchers?	
• Is there a clear description of the research process? (*e.g. step-by-step description of data analysis procedures*)	
**Case purpose**	
• Does the case provide information about common or specific psychotherapy processes?	
• Can the case be compared to and aggregated with other psychotherapy case studies on the basis of its studied phenomenon and formulation?	

Research on epistemic issues in case writing (Kaluzeviciute, 2021) and different forms of scientific thinking in psychoanalytic case studies (Kaluzeviciute & Willemsen, 2020) was also utilised to identify case study components that would help improve therapist clinical decision-making and reflexivity.

For the analysis of more complex research components (e.g. the degree of therapist reflexivity), the purpose-based evaluation will utilise a framework approach, in line with comprehensive and open-ended reviewer responses in ETQS (Evaluation Tool for Qualitative Studies) (Long & Godfrey, 2004) (Table 13). That is to say, the evaluation here is not so much about the presence or absence of information (as in the checklist approach) but the degree to which the information helps the case with its unique purpose, whether it is generalisability or typicality. Therefore, although the purpose-oriented evaluation criteria below encompasses comprehensive questions at a considerable level of generality (in the sense that not all components may be required or relevant for each case study), it nevertheless seeks to engage with each type of purpose-based systematic case study on an individual basis (attending to research or clinical components that are unique to each of type of case study).

It is important to note that, as this is an introductory paper to CaSE, the evaluative framework is still preliminary: it involves some of the core questions that pertain to the nature of all six purpose-based systematic case studies. However, there is a need to develop a more comprehensive and detailed CaSE appraisal framework for each purpose-based systematic case study in the future.

## Using CaSE on published systematic case studies in psychotherapy: an example

To illustrate the use of *CaSE Purpose*-*based Evaluative Framework for Systematic Case Studies*, a case study by Lunn, Daniel, and Poulsen (2016) titled ‘*Psychoanalytic Psychotherapy With a Client With Bulimia Nervosa*’ was selected from the Single Case Archive (SCA) and analysed in Table 14. Based on the core questions associated with the six purpose-based systematic case study types in Table 13(1 to 6), the purpose of Lunn et al.’s (2016) case was identified as *critical* (testing an existing theoretical suggestion).

**Table 14:** Using Case Study Evaluation-tool (CaSE): Lunn et al. (2016)’s case ‘*Psychoanalytic psychotherapy with a client with bulimia nervosa*’

Type of case	The studied phenomenon	Patient data	The clinical discourse	Research	Case purpose
Critical	The studied phenomenon is identified as the treatment of bulimia nervosa. The case tests the need of adapting therapeutic approaches to individual patients on the basis of their specific therapeutic needs and goals rather than providing manualised therapy across the entire clinical population.	A patient was selected from an RCT trial where cognitive behavioural therapy (CBT) was found, on average, more effective than psychoanalytic psychotherapy (PP). However, this patient’s symptoms and context indicated that she may benefit from techniques and principles common to PP, which is why she was chosen for this case study. The case involves a lengthy patient description, including previous diagnosis of anorexia nervosa, binge and purge episodes, early object relations, and childhood-rooted trauma. The case provides substantial information on patient’s psychological context and demographics but does not contain cultural information (this may have been deemed not relevant).	The case contains a detailed description of therapeutic interventions, such as therapeutic containment, reflection, and acknowledgement of unconscious, split-off, or disavowed aspects of patient’s experiences. Therapist observations and clinical decision-making are informed by the theoretical PP principles, particularly in terms of affirming and interpreting patient’s experiences. The effectiveness of therapeutic interventions is described as highly positive: patient has stopped binging and purging and was able to develop a closer relationship with her family.	Several theoretical and research perspectives are explored in order to tailor the most suitable approach for the patient, including attachment styles, mentalization and integrative approaches. One of the authors acted as a therapist, while the two other authors were involved in data analysis; this improved the data triangulation process. Several hypothetical assumptions were made about therapeutic setting and relationship and their suitability for this patient; they are shown to be highly effective and helpful later in the case (e.g. nondirective PP therapy was experienced as more helpful by the patient than directive CBT therapy).	The case demonstrates that insight-oriented, nondirective PP can yield significant successes for patients with bulimia nervosa who also display low reflective functioning and insecure attachments. This case is an important critical follow-up to larger RCT study, which by and large favoured CBT to PP for patients with eating disorders.

Sometimes, case study authors will explicitly define the purpose of their case in the form of research objectives (as was the case in Lunn et al.’s study); this helps identifying which purpose-based questions are most relevant for the evaluation of the case. However, some case studies will require comprehensive analysis in order to identify their purpose (or multiple purposes). As such, it is recommended that CaSE reviewers first assess the degree and manner in which information about the studied phenomenon, patient data, clinical discourse and research are presented before deciding on the case purpose.

Although each purpose-based systematic case study will contribute to different strands of psychotherapy (theory, practice, training, etc.) and focus on different forms of data (e.g. theory testing vs extensive clinical descriptions), the overarching aim across all systematic case studies in psychotherapy is to study local and contingent processes, such as variations in patient symptoms and complexities of the clinical setting. The comprehensive framework approach will therefore allow reviewers to assess the degree of *external validity* in systematic case studies (Barkham & Mellor-Clark, 2003). Furthermore, assessing the case against its purpose will let reviewers determine whether the case achieves its set goals (research objectives and aims). The example below shows that Lunn et al.’s (2016) case is successful in functioning as a critical case as the authors provide relevant, high-quality information about their tested therapeutic conditions.

Finally, it is also possible to use CaSE to gather specific type of systematic case studies for one’s research, practice, training, etc. For example, a CaSE reviewer might want to identify as many *descriptive* case studies focusing on negative therapeutic relationships as possible for their clinical supervision. The reviewer will therefore only need to refer to CaSE questions in Table 13(2) on descriptive cases. If the reviewed cases do not align with the questions in Table 13(2), then they are not suitable for the CaSE reviewer who is looking for “know-how” knowledge and detailed clinical narratives.

## Concluding comments

This paper introduces a novel Case Study Evaluation-tool (CaSE) for systematic case studies in psychotherapy. Unlike most appraisal tools in EBP, CaSE is positioned within purpose-oriented evaluation criteria, in line with the PBE paradigm. CaSE enables reviewers to assess what each systematic case is good for (rather than determining an absolute measure of ‘good’ and ‘bad’ systematic case studies). In order to explicate a purpose-based evaluative framework, six different systematic case study purposes in psychotherapy have been identified: *representative cases* (purpose: typicality), *descriptive cases* (purpose: particularity), *unique cases* (purpose: deviation), *critical cases* (purpose: falsification/confirmation), *exploratory cases* (purpose: hypothesis generation) and *transferable cases* (purpose: generalisability). Each case was linked with an existing epistemological network, such as Iwakabe and Gazzola’s (2009) work on case selection criteria for meta-synthesis. The framework approach includes core questions specific to each purpose-based case study (Table 13 (1–6)). The aim is to assess the external validity and effectiveness of each case study against its set out research objectives and aims. Reviewers are required to perform a comprehensive and open-ended data analysis, as shown in the example in Table 14.

Along with *CaSE Purpose*-*based Evaluative Framework* (Table 13), the paper also developed *CaSE Checklist for Essential Components in Systematic Case Studies* (Table 12). The checklist approach is meant to aid reviewers in assessing the presence or absence of essential case study components, such as the rationale behind choosing the case study method and description of patient’s history. If essential components are missing in a systematic case study, then it may be implied that there is a lack of information, which in turn diminishes the evidential quality of the case. Following broader definitions of validity set out by Levitt et al. (2017) and Truijens et al. (2019), it could be argued that the checklist approach allows for the assessment of (non-quantitative) internal validity in systematic case studies: does the researcher provide sufficient information about the case study design, rationale, research objectives, epistemological/philosophical paradigms, assessment procedures, data analysis, etc., to account for their research conclusions?

It is important to note that this paper is set as an introduction to CaSE; by extension, it is also set as an introduction to research evaluation and appraisal processes for case study researchers in psychotherapy. As such, it was important to provide a step-by-step epistemological rationale and process behind the development of CaSE evaluative framework and checklist. However, this also means that further research needs to be conducted in order to develop the tool. While *CaSE Purpose*-*based Evaluative Framework* involves some of the core questions that pertain to the nature of all six purpose-based systematic case studies, there is a need to develop individual and comprehensive CaSE evaluative frameworks for each of the purpose-based systematic case studies in the future. This line of research is likely to enhance CaSE target audience: clinicians interested in reviewing highly particular clinical narratives will attend to descriptive case study appraisal frameworks; researchers working with qualitative meta-synthesis will find transferable case study appraisal frameworks most relevant to their work; while teachers on psychotherapy and counselling modules may seek out unique case study appraisal frameworks.

Furthermore, although *CaSE Checklist for Essential Components in Systematic Case Studies* and *CaSE Purpose*-*based Evaluative Framework for Systematic Case Studies* are presented in a comprehensive, detailed manner, with definitions and examples that would enable reviewers to have a good grasp of the appraisal process, it is likely that different reviewers may have different interpretations or ideas of what might be ‘substantial’ case study data. This, in part, is due to the methodologically pluralistic nature of the case study genre itself; what is relevant for one case study may not be relevant for another, and vice-versa. To aid with the review process, future research on CaSE should include a comprehensive paper on using the tool. This paper should involve evaluation examples with all six purpose-based systematic case studies, as well as a ‘search’ exercise (using CaSE to assess the relevance of case studies for one’s research, practice, training, etc.).

Finally, further research needs to be developed on how (and, indeed, whether) systematic case studies should be reviewed with specific ‘grades’ or ‘assessments’ that go beyond the qualitative examination in Table 14. This would be particularly significant for the processes of qualitative meta-synthesis and meta-analysis. These research developments will further enhance CaSE tool, and, in turn, enable psychotherapy researchers to appraise their findings within clear, purpose-based evaluative criteria appropriate for systematic case studies.

## Data Availability

Not applicable.
